# Saffron and Its Major Ingredients’ Effect on Colon Cancer Cells with Mismatch Repair Deficiency and Microsatellite Instability

**DOI:** 10.3390/molecules26133855

**Published:** 2021-06-24

**Authors:** Amr Amin, Aaminah Farrukh, Chandraprabha Murali, Akbar Soleimani, Françoise Praz, Grazia Graziani, Hassan Brim, Hassan Ashktorab

**Affiliations:** 1 Biology Department, College of Science, United Arab Emirates University, AI-Ain 15551, United Arab Emirates; a.amin@uaeu.ac.ae (A.A.); 201870123@uaeu.ac.ae (A.F.); chandra_prabha@uaeu.ac.ae (C.M.); 2 The College, The University of Chicago, Chicago, IL 60637, USA; 3 Department of Pathology and Cancer Center, College of Medicine, Howard University College of Medicine, Washington, DC 20059, USA; hooman1350@yahoo.com (A.S.); hbrim@howard.edu (H.B.); 4 INSERM UMR_S 938, Centre de Recherche Saint-Antoine (CRSA), Sorbonne Université and Centre National de la Recherche Scientifique (CNRS), CEDEX 12, 75012 Paris, France; Francoise.Praz@inserm.fr; 5 Department of Systems Medicine, University of Rome Tor Vergata, Via Montpellier 1, 00133 Rome, Italy; graziani@uniroma2.it

**Keywords:** colorectal cancer, saffron, safranal, crocin, HCT116, MLH1, MSH3, DNA damage and repair, apoptosis

## Abstract

Background: Colorectal cancer (CRC) is one of the most common cancers worldwide. One of its subtypes is associated with defective mismatch repair (dMMR) genes. Saffron has many potentially protective roles against colon malignancy. However, these roles in the context of dMMR tumors have not been explored. In this study, we aimed to investigate the effects of saffron and its constituents in CRC cell lines with dMMR. Methods: Saffron crude extracts and specific compounds (safranal and crocin) were used in the human colorectal cancer cell lines HCT116, HCT116+3 (inserted MLH1), HCT116+5 (inserted MSH3), and HCT116+3+5 (inserted MLH1 and MSH3). CDC25b, p-H2AX, TPDP1, and GAPDH were analyzed by Western blot. Proliferation and cytotoxicity were analyzed by MTT. The scratch wound assay was also performed. Results: Saffron crude extracts restricted (up to 70%) the proliferation in colon cells with deficient MMR (HCT116) compared to proficient MMR. The wound healing assay indicates that deficient MMR cells are doing better (up to 90%) than proficient MMR cells when treated with saffron. CDC25b and TDP1 downregulated (up to 20-fold) in proficient MMR cells compared to deficient MMR cells, while p.H2AX was significantly upregulated in both cell types, particularly at >10 mg/mL saffron in a concentration-dependent manner. The reduction in cellular proliferation was accompanied with upregulation of caspase 3 and 7. The major active saffron compounds, safranal and crocin reproduced most of the saffron crude extracts’ effects. Conclusions: Saffron’s anti-proliferative effect is significant in cells with deficient MMR. This novel effect may have therapeutic implications and benefits for MSI CRC patients who are generally not recommended for the 5-fluorouracil-based treatment.

## 1. Introduction

Colorectal cancer (CRC) is one of the leading causes of cancer-related mortality and the third most prevalent cancer among both genders [1,2]. Most CRCs (60–85%) occur sporadically through acquired genetic mutations. About 5% of the cases consist of genetic cancer syndromes such as hereditary non-polyposis CRC (also called Lynch syndrome) caused by defective mismatch repair (MMR) enzymes such as MLH1 [3].

Microsatellite instability (MSI) in sporadic CRC is frequently caused by promoter hyper-methylation of the mismatch repair gene MLH1 and is highly associated with the CpG methylator phenotype (CIMP) [4,5].

The DNA mismatch repair system is an evolutionary conserved process responsible for fixing errors occurring during replication of proliferating cells. MMR is necessary to ensure the stability of the genetic information and to avoid future genetic diseases. This DNA repair machinery is regulated by different proteins such as: H2AX, PARP, TDP1, TOP1, etc. [6]. 

The use of natural compounds as therapeutics especially in combination with chemotherapeutic agents is attractive. There are many benefits of using natural compounds compared to the present-day medicine. Natural compounds have much lesser side effects and are nutritionally beneficial and cost-effective. One of the most studied natural compounds is *Crocus sativus*, commonly known as saffron. Saffron has been used as an analgesic, anti-spasmodic, and anti-depressant agent for quite some time [7]. Saffron and its constituents, especially safranal and crocin, have been studied owing to their therapeutic properties [8,9,10,11,12]. Saffron has reportedly served as an anti-cancer agent by inhibiting the DNA/RNA synthesis of malignant lung tumor cells and inhibiting proliferation of cancerous cells by apoptosis in Hela (human cervical carcinoma) and HepG2 (human hepatocellular carcinoma, HCC) cells [12]. 

Amin et al. have successfully reported the anticancer effect of saffron in mouse models of HCC. On treatment with saffron, the nodule formation of DEN-treated mice had been suppressed. This suppression was correlated with apoptosis and reduced the cell proliferation and oxidative stress [11]. 

**Figure 1:**
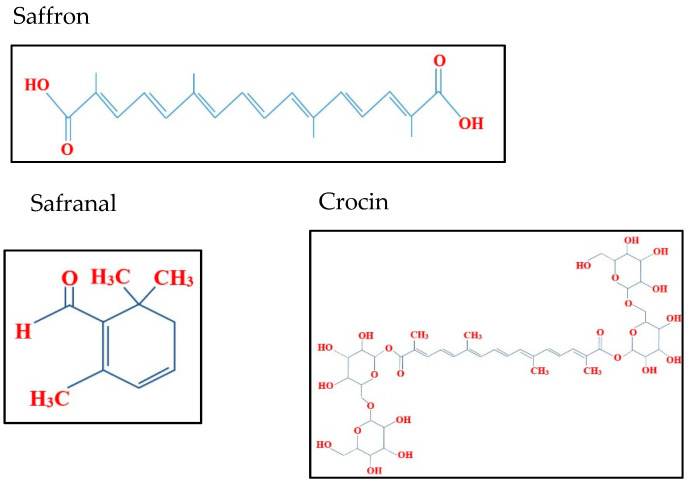
Chemical structure of saffron, safranal and crocin.

Similarly, safranal which is given for the odor of saffron, has been extensively studied for its anti-cancerous and anti-tumor activities. Safranal’s mechanisms of action include DNA double strand breakage (DSBs), induction of apoptosis, and inhibition of epithelial-mesenchymal transition (EMT). Safranal has shown similar pro-apoptotic and anti-proliferative properties in Hela cells, A549 (human lung cancer) and PC-3 (human prostate cancer) cells [8,12]. Crocin, that confers saffron’s bright color, has reported pro-apoptotic properties in Hela and two different human skin cancer cell lines (A431 and SCL-1) [13]. Crocin has also reportedly exhibited selective cytotoxicity against HepG2, HL60 (promyelocytic leukemia), and different CRC cell lines [12]. The chemical structure of Saffron and its constituents, safranal and crocin is shown in Figure 1.

In this study, we assessed saffron and its major components on HCT116 cells. Previously, two similar studies were conducted wherein HCT116 cells with different p53 (a tumor suppressor protein) variants, were subjected to the treatment with crocin and saffron. These studies revealed that saffron and crocin have an anti-proliferative and pro-apoptotic effect and induced cell cycle arrest. Saffron had also resulted in autophagic cell death, but such pro-autophagic effect was not observed on the treatment with crocin [10,14]. The regulation of cell cycle is an important mechanism for cell survival. Abnormalities in this mechanism are frequently reported in most cancers. An important protein in this mechanism is CDC25b, which allows the cell to proceed from G2 to M phase of the cell cycle [8]. CDC25b can serve as an important therapeutic biomarker in research, to evaluate the effectiveness achieved against malignant cells by novel compounds.

Our proposed study is performed using the same cells HCT116 which will be supplemented with the two missing MMR genes (MLH1 and MSH3) to assess saffron and the associated compounds’ effects in the context of an MMR-deficient (dMMR) or -proficient genotype (pMMR).

## 2. Results

### 2.1. Saffron and Crocin Decrease the Viability of pMMR HCT116 Compared to MSH3, MLH1, and Parental HCT116 

**Figure 2:**
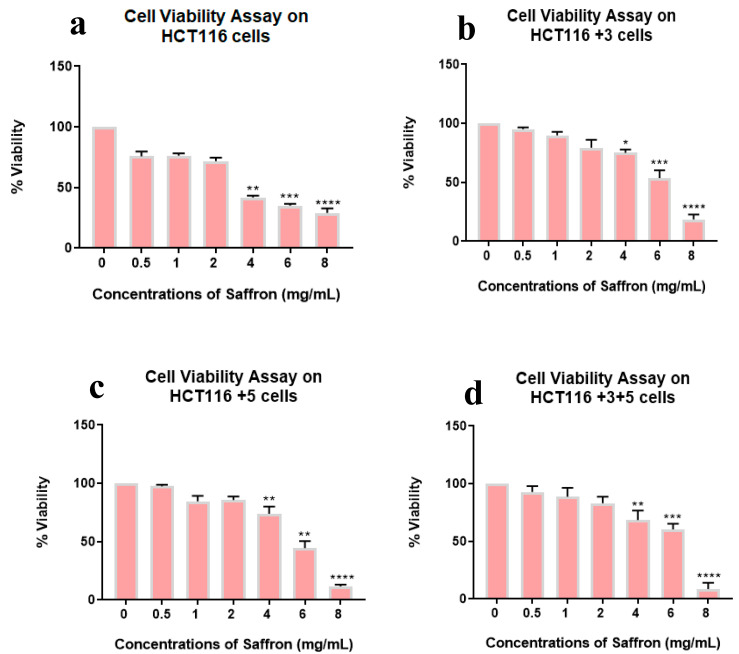
Cell viability assay of saffron-treated cells. Percentage of viability shown vs. concentration of saffron. (**a**–**d**) show the effect of saffron treatment on the viability of HCT116, HCT116+3, HCT116+5 and HCT116+3+5 cells respectively. (* *p* < 0.05, ** *p* < 0.01, *** *p* < 0.001 and **** *p* < 0.0001).

The effect of saffron and its derivatives on the viability of HCT116 cells was determined using a cell viability assay, wherein the cells were treated with 0–8 mg/mL of saffron for 24 h. The whole saffron treatment showed a dose dependent decrease in cell viability in parental HCT116 cells (25 to 70%) compared to +3, +5 or +3+5 cells (Figure 2).

Viability was lowest at 8 mg/mL in all cell types while the gradual viability decrease was observed in HCT116+3+5 then HCT116+3, HCT116+5, and HCT116 parental. This result indicates the impact of MMR genes, MSH3, and MLH1, in the sensitivity of cell viability to the whole saffron treatment.

**Figure 3:**
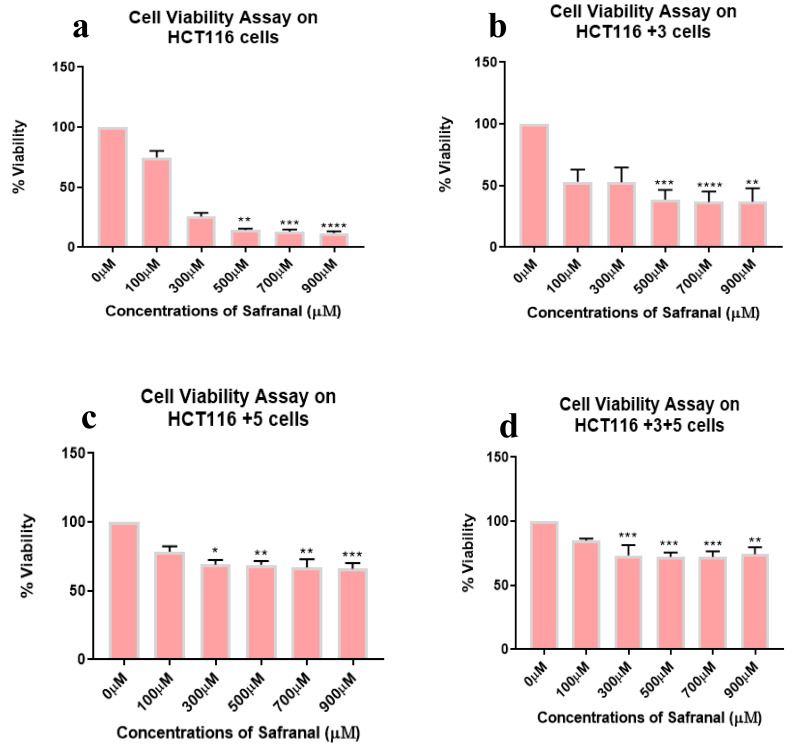
Cell viability assay of safranal-treated cells. Percentage of viability shown vs. concentration of safranal. (**a**–**d**) show the effect of safranal treatment on the viability of HCT116, HCT116+3, HCT116+5 and HCT116+3+5 cells respectively. (* *p* < 0.05, ** *p* < 0.01, *** *p* < 0.001 and **** *p* < 0.0001).

Safranal showed a dose dependent inhibition (0–900 µM) in all cell lines with the most significant effect observed in HCT116 (50–70%) and HCT116 +3 (25–90%) cells at 100 µM (Figure 3).

**Figure 4:**
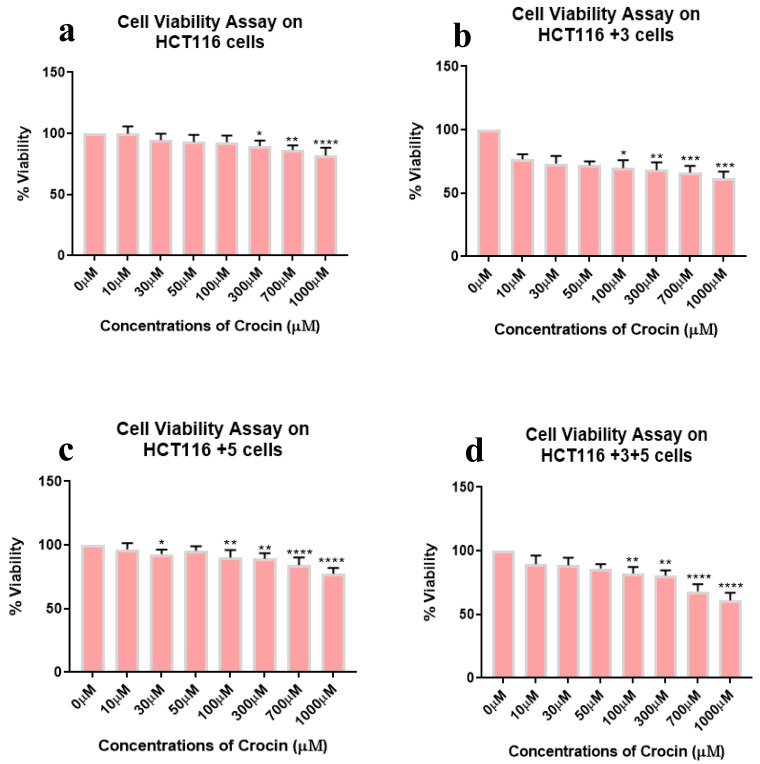
Cell viability assay of crocin-treated cells. Percentage of viability shown vs. concentration of crocin. (**a**–**d**) show the effect of crocin treatment on the viability of HCT116, HCT116+3, HCT116+5 and HCT116+3+5 cells respectively. (* *p* < 0.05, ** *p* < 0.01, *** *p* < 0.001 and **** *p* < 0.0001).

To determine the effect of crocin, cells were treated with different doses, 0–1000 µM for 24 h. Out of all the treated cells, +3 and +3+5 cells showed a significant inhibitory effect in a dose-dependent manner (Figure 4). Viability was lowest at 1000 µM crocin in all cell types while the gradual viability decrease was observed in HCT116+3+5 (10 to 40%) then HCT116+3 (25 to 40%), HCT116+5 (5 to 25%), and HCT116 parental (20% at 1000 µM). This result indicates the impact of MMR genes, MSH3 and MLH1, in sensitivity of cell viability to the crocin treatment. 

### 2.2. Effect of Saffron and Its Derivatives on the Migration of HCT116 Cells and MMR Genes, MSH3, MLH12 Proficient Subtypes

**Figure 5:**
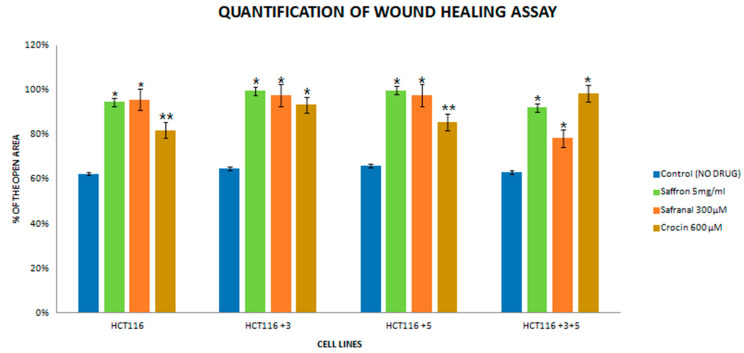
Quantification histogram of the wound healing assay. The assay was performed in triplicates. (* *p* < 0.05, ** *p* < 0.01).

The migration assay provides an important insight in the progression of cancer, hence after validating the effect of saffron and its derivatives on the viability of HCT116 cells, the effect on migration was determined. All cells were treated with the following doses: Saffron 5 mg/mL, safranal 300 µM, and crocin 600 µM for 24 h and compared with the control (Figure 5). The doses were selected conservatively to minimize their effects on the cells’ morphology.

The images were captured at 0 and 24 h for the control and treated cells. For saffron-treated cells, the 0 h image is not available due to the interference of saffron’s bright color. The treatment with saffron showed a significant decrease (90% open wound area) in the migratory and invasive capacity of proficient MMR in HCT116+3+5 cells (Figure 5) compared to the parental HCT-116. Safranal had its most significant effect in +3+5 cells (80% open wound area) where it inhibited the migration of cells considerably 24 h post-treatment compared to the control (Figure 5).

### 2.3. Cell Cycle and DNA Repair Machinery Are Affected by Saffron and Its Derivatives

In order to determine the pathway involved in the cytotoxic nature of saffron and its derivatives, markers of DNA damage and repair and cell cycle were analyzed. Immunoblots of p.H2AX and TDP1 (involved in the repair of stalled Topoisomerase I-DNA complexes) were analyzed for DNA damage and repair. A key cell cycle checkpoint regulator CDC25b was also analyzed.

**Figure 6:**
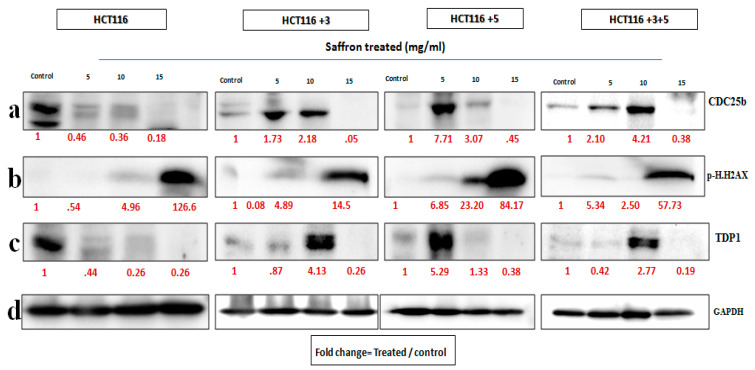
Saffron’s effect on cell cycle and DNA repair machinery [15]. Cells were treated with saffron 5, 10, and 15 mg/mL for 24 h. (**a**–**c**) show the effect of Saffron treatment on CDC25b, p.H2AX and TDP1 respectively. (**d**). GAPDH was used as loading control. Red font represents the fold change value.

To determine the effect of saffron, cells were treated with 5, 10, and 15 mg/mL of saffron for 24 h. Expression of CDC25b decreased at 15 mg/mL of saffron in mutant cells but not consistent in a dose dependent manner (Figure 6). The DDR markers, p.H2AX and TDP1 showed an increase and decrease in protein expression, respectively at a dose of 15 mg/mL saffron in all cell lines (Figure 6). The fold change of relative expression compared to the control is mentioned below each band.

**Figure 7:**
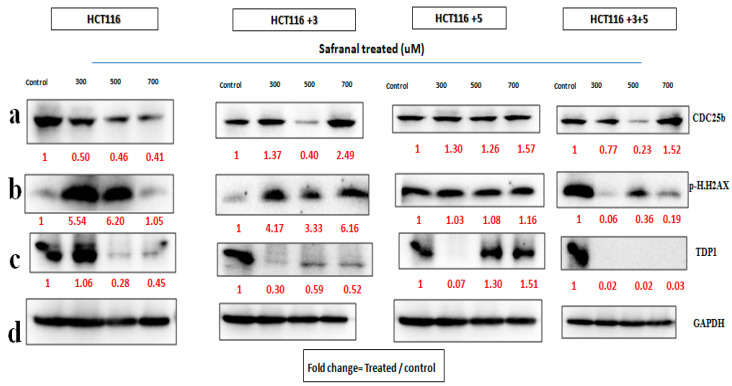
Safranal’s effect on cell cycle and DNA repair machinery. (**a**–**d**) Cells were treated with safranal 300, 500, and 700 µM for 24 h. (**a**–**c**) show the effect of Safranal treatment on CDC25b, p.H2AX and TDP1 respectively. (**d**). GAPDH was used as loading control. Red font represents the fold change value.

For safranal’s treatment, the cells were treated with 300, 500, and 700 µM for 24 h (Figure 7). The expression of CDC25b decreased with the increase in safranal concentration for parental HCT116 cells. There was a relative decrease in protein expression in +3 and +3+5 cells, compared to the control at 500 µM. The expression of p.H2AX increased in HCT116 cells at 300 and 500 µM, and in HCT116+3 cells at 300 and 700 µM TDP1 expression decreased at the 500 µM treatment of HCT116 cells, at 300 µM in +3 and +5 cells, and constantly decreased with all doses in HCT116+3+5 cells (Figure 7). The fold change of the relative expression compared to the control is mentioned below each band.

**Figure 8:**
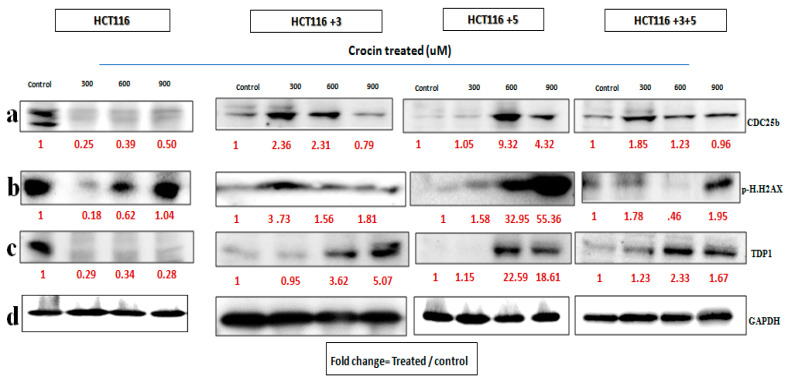
Crocin’s effect on cell cycle and DNA repair machinery. (**a**–**d**) Cells were treated with crocin 300, 600, and 900 µM for 24 h. (**a**–**c**) show the effect of Crocin treatment on CDC25b, p.H2AX and TDP1 respectively. (**d**). GAPDH was used as loading control. Red font represents the fold change value.

To evaluate the effects of crocin, the cells were treated with 300, 600, and 900 µM crocin for 24 h. The CDC25b expression in HCT116 cells on the treatment with 300 µM and in +3 cells on the treatment with 900 µM was decreased. The p.H2AX expression increased on the treatment with 600 µM in +3 cells and on the treatment with 600 and 900 µM in +5 cells (Figure 8). The fold change of the relative expression compared to the control is mentioned below each band.

To sum up, the expression of CDC25b was completely downregulated in both HCT116 parental and mutant cells at a dose of 15 mg/mL saffron. Safranal and crocin were not as consistent on the CDC25b protein, with crocin showing a steady effect only on the HCT116 parental. In the HCT116 parental, the protein levels of p.H.H2AX were upregulated in a dose dependent manner by saffron (15 mg/mL), safranal (300 and 500 μM), and crocin (600 and 900 μM) at the stated doses. Crocin (900 μM) had a persistent upregulation of p.H2AX in mutant cells where as safranal and saffron showed irregular results. Meanwhile, crocin, saffron, and safranal inhibited the TDP1 protein in parental HCT116 cells in a dose dependent manner. Safranal reduced the expression of TDP1 in HCT116+3 and HCT116+3+5 cells, whereas, crocin and saffron did not have a constant effect on TDP1 in mutant cells.

### 2.4. Saffron, Crocin, and Safranal Induce Apoptosis

**Figure 9:**
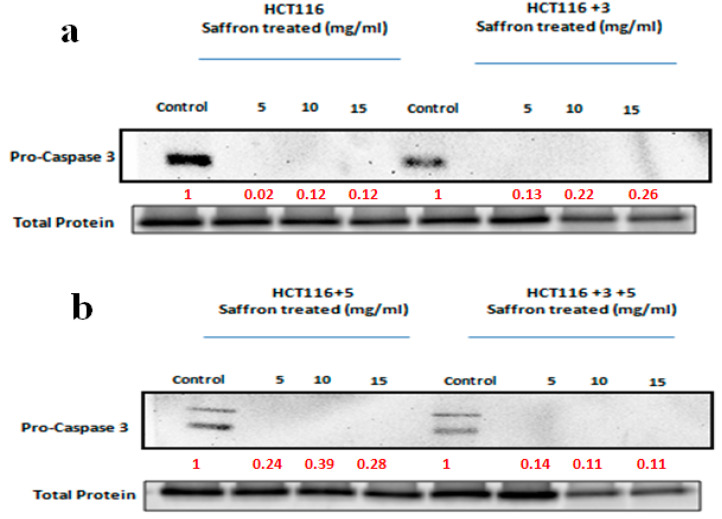
Saffron activates the caspase pathway. A Western blot analysis was performed to determine the expression of pro-caspase 3 in saffron-treated cells. The indicated cells were treated with 5, 10, and 15 mg/mL of saffron for 24 h. Pro-caspase 3, which cleaves to caspase 3, was analyzed. The saffron treatment led to a decrease in the expression of pro-caspase 3, which was visible in the control cells. The fold change of relative expression compared to the control is mentioned below each band. Red font represents the fold change value. (**a**,**b**) show the effect of saffron treatment on caspase pathway in HCT116, HCT116+3 and HCT116+5, HCT116+3+5 cells respectively.

The effect of saffron and its compounds on apoptosis was analyzed. The activity and expression of caspase 3 and 7 were analyzed, since they are the executioner caspases of apoptosis. A Western blot analysis was performed to determine the expression of pro-caspase 3 in saffron-treated cells (Figure 9). For safranal and crocin-treated cells, the caspase 3/7 activity was also measured. The caspase activity for saffron-treated cells was not measured since saffron’s color interfered with the assay.

To study the effect of saffron on apoptosis, cells were treated with 5, 10, and 15 mg/mL of saffron for 24 h (Figure 9). Pro-caspase 3, which cleaves to caspase 3, was analyzed. The saffron treatment led to a decrease in the expression of pro-caspase 3, which was visible in the control cells. The fold change of the relative expression compared to the control is mentioned below each band.

**Figure 10:**
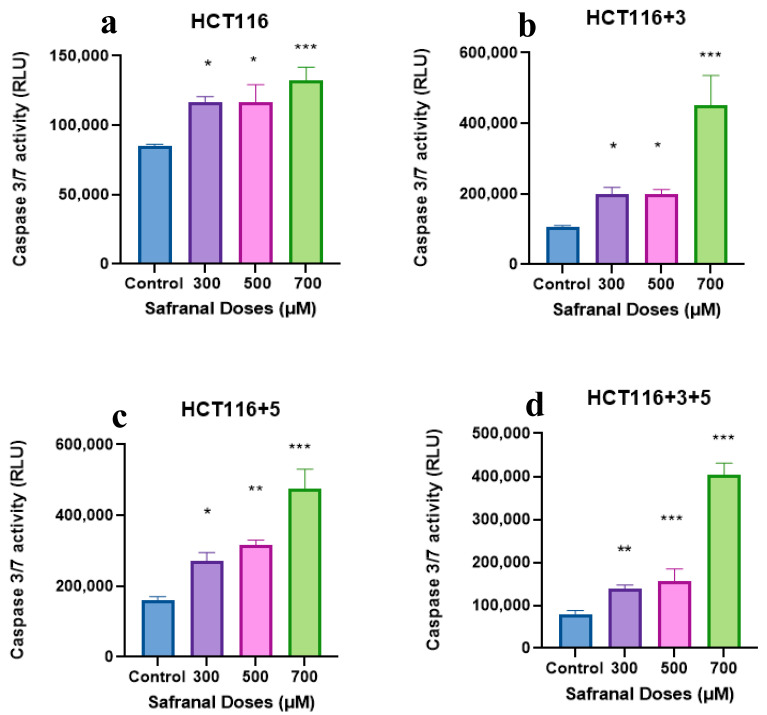
Effect of safranal on the caspase pathway (caspase activity measured in the relative light unit (RLU). (**a**–**d**) show the effect of Safranal on the caspase activity in HCT116, HCT116+3, HCT116+5 and HCT116+3+5 cells respectively. An ANOVA (Analysis of Variance) test was carried out (≥0.05 NS, ≤0.01 *, <0.01 **, ≤0.001 ***).

The activity of executioner caspases, caspase 3 and 7, was measured after treating the cells with safranal 300, 500, and 700 µM (Figure 10). All cell lines showed an increase in caspase activity with the increase in dose, with the most significant effect observed at 700 µM compared to the control. Safranal showed an increased caspase 3/7 activity in HCT116+3+5 (approx. 300-fold), HCT116+3 (approx. 400-fold) then HCT116+5 (approx. 200-fold). These results suggest that MSH3 and MLH1 play an important role in processes involving saffron and apoptosis.

**Figure 11:**
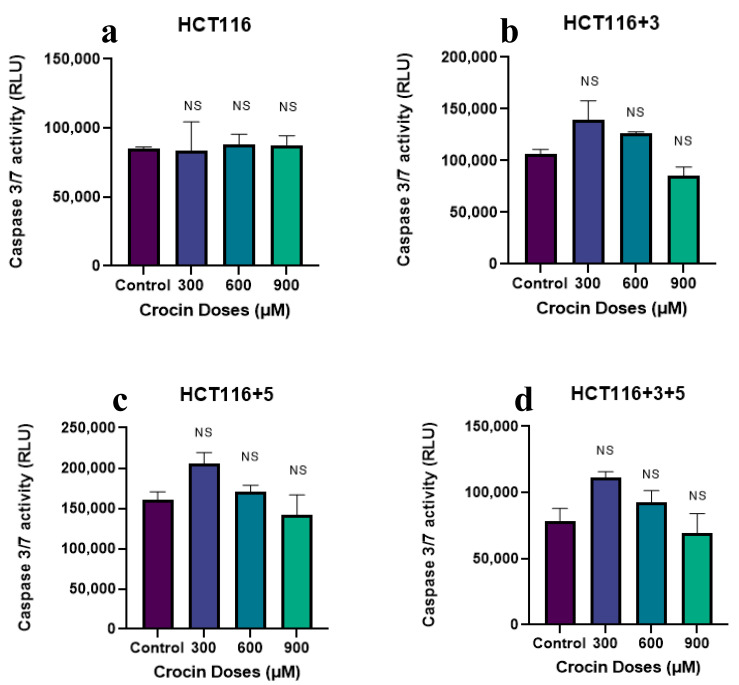
Effect of crocin on caspase pathway (caspase activity measured in the relative light unit (RLU). (**a**–**d)** show the effect of crocin on the caspase activity in HCT116, HCT116+3, HCT116+5 and HCT116+3+5 cells respectively. An ANOVA (Analysis of Variance) test was carried out (≥0.05 NS).

To evaluate the effect of crocin, cells were treated with 300, 600, and 900 µM following the protocol. The cells showed an increase in caspase activity on the treatment with 300 µM in HCT116 +3, HCT116 +5, and HCT116+3+5 cells (approx. 50-fold) (Figure 11). The effect was not consistent and significant when compared to the same as imparted by safranal on these cells.

## 3. Discussion

Colorectal cancer is one of the leading causes of cancer-related deaths worldwide. Different environmental and genetic factors are responsible for its development. Different CRC subtypes such as MSI and microsatellite stable (MSS) tumors may have different outcomes due to their different molecular and pathological characteristics. Patients with MSI tumors have better prognosis than MSS ones. While those with EMAST tumors have different immunological and pathological features and poor prognosis, as well. The CRC treatment based on MMR deficiency is a key approach for better outcome and the need for healthier and long-lasting treatments remain pivotal [16]. In this study, we showed that saffron’s anti-proliferative effect is significant in cells with deficient MMR.

Saffron has long been used as a traditional medicine. It has shown anti-tumor properties in vitro in different cancer cell lines such as those from liver and prostate. The therapeutic efficacy of saffron has also been observed with its derivatives: Safranal and crocin. Saffron and its derivatives have shown no detrimental effects on normal cells, hence making it a model curative agent that can be used as an adjuvant therapy [8]. Indeed, we recently showed that it can affect the action of MACC1, a metastasis-associated gene in CRC [17]. In the present study, we analyzed its effects within the context of MMR genes deficiency in HCT116 cell lines.

HCT116 cells with different MLH1 and/or MSH3 gene supplements were treated with different doses of saffron, ranging from 0–10 mg/mL for 24 h, to assess cell viability. All cells had shown significant dose-dependent inhibition on the treatment with saffron (Figure 2). This dose-dependent inhibition is consistent with the results previously published by Bajbouj et al. where HCT116 cells (with or without p53) were studied for saffron’s effect [14]. Saffron caused DNA damage and promoted apoptosis in both tested cell lines. An interesting observation was the delayed apoptosis in cells that lacked p53 [14]. Saffron has previously been reported to inhibit cell viability at lower doses (100–150 µg/mL) in other cancer cell lines, suggesting that HCT116 cells have higher resistance to this compound [12]. On treatment with safranal and crocin, the cells exhibited a dose-dependent cell viability inhibition pattern, similar to saffron (Figure 3 and Figure 4). Safranal and crocin dose ranges in our study are close to the effective doses published for other cancers [9,18,19]. Malaekeh-Nikouei et al. have reported safranal’s effective dose range to be much higher for other cells suggesting that HCT116 cells have a higher susceptibility [20]. Cancer cells migration and invasion are critical features for metastasis [21]. Saffron, safranal, and crocin treatments showed a significant decrease in migration as compared to the control. Safranal has been previously shown to have anti-migration properties on other cell lines including HepG2 (liver cancer cells) and HSC-3 (prostate cancer cells). Although the effective concentration for HSC-3 cells was lower, the concentration is closer to what is reported for HepG2 cells [8]. The results of crocin treatment are also consistent with the previous findings where the crocin treatment has inhibited invasion of different cell lines including HUVEC (human umbilical vein endothelial cells), AGS and HGS-27 (gastric cancer cells), and MG63 (osteosarcoma) [22,23]. Interestingly, Wang et al. reported crocin’s inability to inhibit the invasion of HCT116 cells, at 271.18 µM, but comparing it with the present findings suggests that a higher dose is required to inhibit invasiveness (Figure 5) [19]. In patients with CRC, recurrence and metastasis are the most common causes of cancer-related death. The ability of saffron and its derivatives to minimize migration of HCT116 cells provides a novel course of action that can be used as an adjuvant therapy, especially in patients with MSI tumors.

In order to determine the effect of treatment on the cells, with altered MLH1 and/or MSH3 genes, immunoblots of two DNA damage markers, p.H2AX and TDP1, together with CDC25b, a cell cycle checkpoint regulator, were analyzed (Figure 6, Figure 7 and Figure 8). To our knowledge, the effect of crocin on these particular markers is reported here for the first time, making it a novel finding in the saffron mechanistic action.

As mentioned earlier, DNA MMR is required to ensure genetic stability. A commonly occurring error in DNA replication is DNA DSBs. H2AX, a histone, is phosphorylated to p.H2AX in response to DSB and hence is a marker for studying DNA damage [8]. In this study, saffron and its derivatives upregulated the expression of p.H2AX. Saffron up regulated the expression at 15 mg/mL across all the different cells (Figure 6), indicating a DNA damage as a result of the treatment. Similar findings were also reported by Bajbouj et al. where the treatment with saffron had shown increased expression of p.H2AX in HCT116 cells [14]. p.H2AX was also reported to have been induced by saffron at a lower dose, 6 mg/mL, in HepG2 cells [8]. Safranal upregulated the expression in HCT116 and HCT116 +3 cells, compared to the untreated control (Figure 7). The effective concentrations observed in this study are closer to a previously mentioned effective concentration, where safranal at 500 µM had similar effects on HepG2 cells [8].

TDP1, such as p.H2AX, is involved in the DNA repair. It protects against DNA strands breakage by repairing stalled Topoisomerase I-DNA complexes, occurring as a result of DSBs [8]. All cells treated with saffron and its derivatives displayed downregulated the TDP-1 expression. The most notable response for saffron treated cells was observed at 15 mg/mL (Figure 6). This downregulation of TDP-1 is likely the result of a cellular decision to allow such DNA-damaged cells to undergo apoptosis rather than repairing them.

In the case of damage to DNA, the cell cycle comes to an arrest and induces apoptosis or cell death. After establishing the effect of treatment on DNA damage, its effect on cell cycle was evaluated. CDC25b, is a key cell cycle regulator and also an oncogene with an over-expression observed in breast cancer, lung cancer, and CRC [6]. Saffron showed a dose-dependent inhibition of CDC25b in HCT116 cells while for the other cells an inhibition was observed at 15 mg/m. Doxorubicin, a widely used chemotherapeutic agent, had no effect on CDC25b in HCT116 cells [24], while saffron inhibited the expression of CDC25b. The treatment with safranal showed a similar pattern of dose dependent inhibition, for HCT116 cells (Figure 7). The treatment with crocin showed varying results, but overall, the expression was downregulated as compared to the untreated controls.

Cells undergo apoptosis in the case of irreparable damage. Our results indicated DNA damage and cell cycle arrest in response to the saffron treatment. To further confirm the apoptotic property of saffron, executioner caspases of apoptosis, caspase 3 and 7, were studied. Immunoblots for saffron-treated cells showed the absence of pro-caspase 3, suggesting its cleavage to its active form (Figure 9). Saffron has also exhibited such pro-apoptotic properties in liver and breast cancer cells [25,26]. The activity of caspase 3 and 7 for safranal and crocin-treated cells was measured using the Caspase-Glo^®^ 3/7 Assay kit (Figure 10. Effect of safranal on the caspase pathway (caspase activity measured in the relative light unit (RLU). An ANOVA (Analysis of Variance) test was carried out (≥0.05 NS, ≤0.01 *, ≤0.001 ***).

Both saffron compounds showed a significant increase of caspase activity post-treatment compared to the control suggesting an increase in apoptotic activity. Safranal has also shown such caspase activity, previously, on HepG2 cells. These findings are in concordance with the previously available data where safranal has shown similar pro-apoptotic properties in A549 (alveolar human lung cancer) and PC-3 (human prostate cancer) cell lines, as reported by Al-Hrout et al. [8]. Crocin has previously displayed pro-apoptotic properties across different cell lines, including liver, breast, skin, and gastric cancer cells [13,22,27,28] but the effective dose was lower compared to the one used in this study.

## 4. Materials and Methods

### 4.1. Cell Lines

The isogenic HCT116 cell line that is deficient in both MLH1 and MSH3 genes was used in this study [29,30]. This cell line was complemented for these MMR gene deficiencies by transfecting chromosomes 3 and 5 separately to create HCT116+3 and HCT116+5 and together to create HCT116+3+5 cell lines. Briefly, MSH3-deficient/MLH1-proficient colorectal cancer HCT116 (MLH1) cells were transfected with the MLH1 cDNA cloned into the pcDNA3.1 (−) vector [30]. MSH3/MLH1-deficient HCT116, carrying MLH1 and MSH3 mutations on chromosome 3 and 5, respectively, and HCT116 in which parental MLH1 (HCT116 +3), MSH3 (HCT116 +5) or both genes (HCT116 +3+5) were introduced by the chromosome transfer by the microcell transfer of normal human chromosome 3 and/or 5 (Table 1) [29,30,31].

### 4.2. Cell Cultures

The HCT116 cells were cultured in the RPMI 1640 Medium (Hyclone, Marlborough, MA, USA) while HCT116 +3, HCT116 +5, and HCT116 +3+5 cells were cultured in McCoy’s Medium (Hyclone, Marlborough, MA, USA). Both media were supplemented with 10% fetal bovine serum (Sigma Aldrich, St. Louis, MO, USA) and containing 1% of 100 U/mL penicillin and 100 µg/mL streptomycin (Sigma Aldrich, St. Louis, MO, USA) at 37 °C in a humidified 5% CO2 atmosphere. Cells were sub-cultured every 3–5 days using Trypsin 0.25% (Hyclone, Marlborough, MA, USA).

**Saffron, safranal, and crocin preparations:** Saffron crude extracts (Gulf Pearls SPRL Brussels, Belgium, www.gp-food.com, accessed on 3 May 2021) and specific compounds (safranal and crocin (Sigma Aldrich, St. Louis, MO, USA)) were used as previously described [7,17]. Saffron was grinded by a pestle and mortar and dissolved in water at a final concentration of 20 mg/mL and mixed on an orbital shaker in the dark for 1 h then used at different concentrations.

### 4.3. Cell Viability Assay

All cells were seeded at a density of 5000 cells/well in 96-well plates in 100 µL of complete growth medium. Cells were allowed to attach for 24 h before being treated with different concentrations of saffron, safranal, and crocin for 24 h. Cells were then treated with 3-[4,5-dimethylthiazol-2-yl]-2,5-diphenyltratrazolium bromide (MTT) (Sigma Aldrich, St. Louis, MO, USA) and incubated for 3 h. The formed formazan crystals were dissolved using DMSO (Sigma Aldrich, St. Louis, MO, USA) and the absorbance of the resulting product was measured at 570 nm using GloMax Microplate Reader (Promega, Madison, WI, USA). Cell viability is presented as the percent of viable cells = (Abs. of treated cells/Abs. of control cells) × 100. The experiment was carried out in triplicates.

### 4.4. Wound Healing Assay

For the wound healing assay, cells were seeded in 6-well plates. When cells were confluent, a scratch was made with a 200 µL sterile pipette tip, generating a cell-free area of approximately 1 mm in width. Cellular debris was removed by gentle washing with the culture medium and the photos of the wounds (0 h) were taken. Afterwards, the medium was replaced by the culture medium with different concentrations of the drugs and the cells were allowed to migrate for 24 h. At the end of migration experiment, another set of photos was taken, from the same regions using Phase Contrast Fluorescence microscopy (Olympus, Tokyo, Japan). The gap size was analyzed using the Image-J software (LOCI, Madison, WI, USA). To assess the migration ability of cultured cells, cell-free areas of the scratches at 24 h after scratching were subtracted from the area of the scratches at 0 h and calculated as a percentage of untreated (0 h) cultures. The experiment was carried out in triplicates.

### 4.5. Caspase 3 and 7 Activity

Caspase 3 and 7 activities were detected using the Caspase-Glo^®^ 3/7 Assay kit (Promega, Madison, WI, USA) following the manufacturer’s protocol. Briefly, the cells were seeded at a density of 5000 cells/well in 100 µL media for 24 h. Next, the cells were treated with saffron (0.5–8 mg/mL), crocin (100–900 µM) or safranal (10–1000 µM) for 24 h. The next day, the Caspase-Glo reagent (Promega, Madison, WI, USA) was added to the cells and incubated for 4 h. The control which was composed of untreated cells and a blank control, containing media with the reagent, was also included to subtract the background. The luminescence was measured using the GloMax Microplate Reader (Promega, Madison, WI, USA).

### 4.6. Western Blot

Cells were seeded at a density of 1 × 10^6^ cells/100 mm plate and allowed to attach before being treated with saffron, safranal, and crocin for 24 h. Whole cell extracts were isolated using the RIPA buffer (Sigma Aldrich, St. Louis, MO, USA) and protease inhibitors (Sigma Aldrich, St. Louis, MO, USA). The concentration of the isolated proteins was determined using the BCA Protein Assay Reagent (Bio-Rad, Hercules, CA, USA). Thirty micrograms of protein were separated using 10–15% SDS Polyacrylamide gel electrophoresis in our lab. Proteins were transferred onto PVDF membranes (Bio-Rad, Hercules, CA, USA). Membranes were then blocked using 5% BSA (Bovine Serum Albumin) (Sigma Aldrich, St. Louis, MO, USA) followed by incubation with various primary antibodies against CDC25b, p.H2AX, TDP1, and Pro-caspase 3 (1:2000) (Cell Signaling Technology, Danvers, MA, USA) and appropriate secondary antibodies, HRP conjugated (Cell Signaling Technology, Danvers, MA, USA). GAPDH (1:10,000) (Abcam, Cambridge, UK) was used as the loading control. Protein bands were detected using the Westernsure Chemiluminescent Substrate (LI-COR, Lincoln, NE, USA). The experiment was carried out in triplicates.

### 4.7. Statistical Analysis

An ANOVA (Analysis of Variance) test was carried out (<0.05). All statistical analyses were conducted using the GraphPad Prism 8 software (San Diego, CA, USA).

## 5. Conclusions

In this study, the colorectal cell line HCT116 and derivatives with MLH1 and/or MSH3 added genes were treated with saffron and its main compounds, safranal and crocin. The treatment inhibited cell proliferation and blocked cell invasion. On further investigation, it was deduced that the mechanism of action for inhibiting the proliferation was by modulating the cell cycle and DNA damage and repair system. The treatment had also manifested pro-apoptotic properties. It is noteworthy, that the different cells did not respond in a particular manner to the different treatments conducted, nor had the compounds expressed any pattern for a particular cell type. The differential effects of saffron and its main components safranal and crocin in the different tested cell lines, might be capitalized on to develop specific compound protocols in patients with different types of genetic instability in their tumors. In vivo testing in mice models of MSI and MSS CRC is needed to potentially translate these findings in human subjects with such tumors. If confirmed, saffron as an adjuvant therapy might even be recommended for patients’ MSI tumors regardless of the nature of the primary cancer.

## Figures and Tables

**Table 1:** HCT-116 with and without MMR genes and the corresponding phenotypes.

CRC Cell Line	MLH1	MSH3	Tumor Type
HCT116	−	−	MSI and EMAST
HCT116 +3	+	−	EMAST
HCT116 +5	−	+	MSI with no EMAST
HCT116 +3+5	+	+	MSS

MSI: Microsatellite instable; EMAST: Elevated microsatellite alterations at selected tetranucleotide repeat; MSS: microsatellite stable).

## Data Availability

All data are presented in this study.
